# Supplementary material for: Increased risk of cardiac arrhythmia in Hailey-Hailey disease patients
Source: PLoS One. 2024 Sep 6;19(9):e0309482. doi: 10.1371/journal.pone.0309482 (PMC11379163; doi:10.1371/journal.pone.0309482)
Supplement: S1 Table — A total of 342 individuals with an ICD-10 diagnosis of HHD (Q82.8E) were identified and matched with comparison individuals without HHD on a 1:100 ratio, randomly selected from the general Swedish population (all individuals with HHD diagnosis since the start of the register included). Successful matching was performed for birth year, sex, and county of residence at the time of the first HHD diagnosis of the individual. This matching scheme is referred to as incidence density sampling. Conditional logistic regression analyses were performed for the associations between HHD and the major ICD groups of heart diseases: myocardial infarction [I21], heart failure [I42, I50], and arrhythmias. SAS 9.3 software (SAS Institute, Cary, NC) was used for statistical analyses. The results were expressed as odds ratios and corresponding 95% confidence intervals. As a result of the incidence density sampling, odds ratios can be interpreted as risk ratios (RR). The table also shows the mean age and standard deviation (SD) at first arrhythmia diagnosis among individuals with HHD and comparison individuals. *Diagnoses included in arrhythmias: paroxysmal tachycardia [I47], atrial fibrillation and flutter [I48], and other cardiac arrhythmias [I49]. Within the group called other cardiac arrhythmias, there are ten arrhythmias, including ventricular fibrillation [I49.01], ventricular flutter [I49.02], atrial premature depolarization [I49.1], junctional premature polarization [I49.2], ventricular premature polarization [I49.3], unspecified premature polarization [I49.40], other premature polarization [I49.49], sick sinus syndrome [I49.5], other specified cardiac arrhythmias [I49.8] and cardiac arrhythmia, unspecified [I49.9]. (DOCX) [file pone.0309482.s001.docx]

|  | **Myocardial infarction** | | **Heart failure** | | **Arrhythmia*** | | |
| --- | --- | --- | --- | --- | --- | --- | --- |
|  | N (%) | RR (CI) | N (%) | RR (CI) | N (%) | RR (CI) | Mean age in years (CI) at first diagnosis |
| Individuals with HHD, N = 342 | 20  (5.9) | 1.1 (0.6-1.8) | 20  (5.9) | 1.1  (0.6-1.7) | 40 (11.7) | 1.4  (1.0-2.0) | 67.7  (63.5:72.0) |
| Comparison individuals without HHD, N=34,200 | 1,917 (5.6) |  | 1,917 (5.6) |  | 3,047  (8.9) |  | 71.6.0  (71.5;71.6) |
