# Supplementary material for: Increased risk of cardiac arrhythmia in Hailey-Hailey disease patients
Source: PLoS One. 2024 Sep 6;19(9):e0309482. doi: 10.1371/journal.pone.0309482 (PMC11379163; doi:10.1371/journal.pone.0309482)
Supplement: S2 Table — Inclusion criteria were a diagnosis of HHD set by a dermatologist based on typical clinical appearance, histopathology, and family history. Exclusion criteria were age <18 years, current pregnancy, active substance abuse, and acute illness in the past 4 weeks. The control group was matched for age, sex, and BMI. *After multiple comparisons with Bonferroni post hoc correction, none of the p-values are significant (p<0.005). ** M = males, F = females. (DOCX) [file pone.0309482.s002.docx]

| **Variable** | **Sample size** | | **Mean ± standard deviation** | | **Unpaired t-Test** |
| --- | --- | --- | --- | --- | --- |
|  | HHD | Control | HHD | Control | P-value |
| **Age** | 23 | 23 | 53.6 ± 10  (33 – 75) | 51.5 ± 10.9  (33 – 70) | 0.51 |
| **Sex** | 23 (7M, 16F)** | 23 (7M, 16F)** |  |  |  |
| **ECG parameters** |  |  |  |  |  |
| Heart rate (per minute) | 23 | 23 | 63.30 ± 11.060 (45-85) | 63.21 ± 10.023 (47-84) | 0.977 |
| PQ - interval (ms) | 23 | 23 | 157.66 ± 20.482 (116-194) | 155 ± 24.462 (108-210) | 0.695 |
| QRS - duration (ms) | 23 | 23 | 92 ± 10.531 (78-112) | 92.35 ± 9.810 (74-114) | 0.906 |
| QT - interval (ms) | 23 | 23 | 411.57 ± 30.960 (348-498) | 413.30 ± 28.855 (354-470) | 0.845 |
| QTc -interval (ms)  Bazzet formula | 23 | 23 | 417.74 ± 20.633 (379-466) | 420.61 ± 24.062 (368 - 455) | 0.666 |
| QTc-interval (ms)  Fridericia formula | 23 | 23 | 416.10 ± 31.52  (388 – 456) | 418.42 ± 20.86  (378 – 448) | 0.301 |
| QTc–interval (ms)  Framingham formula | 23 | 23 | 414.99 ± 38.99  (385 – 451) | 417.65 ± 21.65  (372 – 448) | 0.649 |
| QTc – interval (ms)  Hodges formula | 23 | 23 | 414.35 ± 36.74  (387 – 474) | 418.24 ± 19.57  (382 – 456) | 0.333 |
| **Blood biomarkers and electrolytes** |  |  |  |  |  |
| NT-proBNP (ng/L) | 23 | 23 | 66.43 ± 55.717 (6-230) | 94.09 ± 191.369 (11-954) | 0.509 |
| Troponin T (ng/L) | 23 | 23 | 7.83 ± 10.360 (5-55) | 5.26 ± 0.619 (5-7) | 0.845 |
| Sodium | 23 | 23 | 140.87 ± 1.817 (138-145) | 140.43 ± 1.805 (136-144) | 0.420 |
| Potassium | 23 | 23 | 4.09 ± 0.251 (3.7-4.6) | 4.02 ± 0.332 (3.4-4.8) | 0.399 |
| Calcium | 23 | 23 | 2.39 ± 0.090 (2.22-2.58) | 2.34 ± 0.071 (2.22-2.49) | 0.037* |
